# Association of Angiopoietin-2 and Ki-67 Expression with Vascular Density and Sunitinib Response in Metastatic Renal Cell Carcinoma

**DOI:** 10.1371/journal.pone.0153745

**Published:** 2016-04-21

**Authors:** Juhana Rautiola, Anita Lampinen, Tuomas Mirtti, Ari Ristimäki, Heikki Joensuu, Petri Bono, Pipsa Saharinen

**Affiliations:** 1 Comprehensive Cancer Center, Helsinki University Hospital, P.O.B. 180, 00029 HUS, Finland and University of Helsinki, Finland; 2 Translational Cancer Biology Program, Research Programs Unit, and Department of Virology, Haartman Institute, Biomedicum Helsinki, Haartmaninkatu 8, P.O.B. 63, FI-00014, University of Helsinki, Finland; 3 Wihuri Research Institute, Biomedicum Helsinki, Haartmaninkatu 8, FI-00290, Helsinki, Finland; 4 Institute for Molecular Medicine Finland, Haartmaninkatu 8, P.O.B. 63, FI-00014, University of Helsinki, Finland; 5 Pathology, Research Programs Unit and HUSLAB, University of Helsinki and Helsinki University Hospital, P.O.B. 400, FI-00029, HUS, Helsinki, Finland; Beth Israel Deaconess Medical Center, UNITED STATES

## Abstract

The Angiopoietin-2 (Ang2, Angpt2) growth factor is a context-dependent antagonist/agonist ligand of the endothelial Tie2 receptor tyrosine kinase and known to promote tumour angiogenesis and metastasis. Angiopoietin antagonists have been tested in clinical cancer trials in combination with VEGF-based anti-angiogenic therapy, including sunitinib, which is widely used as a first-line therapy for metastatic renal cell carcinoma (mRCC). However, little is known about Ang2 protein expression in human tumours and the correlation of tumour Ang2 expression with tumour vascularization, tumour cell proliferation and response to anti-angiogenic therapies. Here, we evaluated, using immunohistochemistry, the expression of Ang2, CD31 and the cell proliferation marker Ki-67 in the primary kidney cancer from 136 mRCC patients, who received first-line sunitinib after nephrectomy. Ang2 protein expression was restrained to RCC tumour vessels, and correlated with tumour vascularization and response to sunitinib. High pre-therapeutic Ang2 expression, and more strongly, combined high expression of both Ang2 and CD31, were associated with a high clinical benefit rate (CBR). Low cancer Ki-67 expression, but not Ang2 or CD31 expression, was associated with favourable progression-free (PFS) and overall survival (OS) as compared to patients with high Ki-67 expression (PFS 6.5 vs. 10.6 months, *P* = 0.009; OS, 15.7 vs. 28.5 months, *P* = 0.015). In summary, in this study to investigate endothelial Ang2 in mRCC patients treated with first-line sunitinib, high cancer Ang2 expression was associated with the CBR, but not PFS or OS, whereas low Ki-67 expression was significantly associated with long PFS and OS.

## Introduction

Renal cell carcinoma (RCC) represents approximately two to three per cent of adult malignancies worldwide, and has an increasing rate of incidence in many countries [1]. The majority of RCCs are classified as clear cell (80%) and papillary cancers (10%), which are thought to arise in the epithelium of the proximal tubules [2]. In approximately 60–75% of sporadic clear cell RCCs (ccRCC), the von Hippel-Lindau (VHL) tumour suppressor gene is inactivated, resulting in stabilization of the hypoxia inducible factors (HIFs), which regulate metabolic and vascular tumour responses, including increased expression of the HIF target vascular endothelial growth factor (VEGF) [3–5].

The prognosis of metastatic RCC (mRCC) is poor, and the disease is notoriously resistant to chemotherapy, with a minority of the patients responding to traditional immunotherapy, such as interferon and interleukin-2 [1,6]. Since 2005, seven novel targeted therapies inhibiting the VEGF signalling pathway and the mammalian target of rapamycin (mTOR) have been approved for the treatment of mRCC, but complete durable responses with any of the targeted therapies are rare [7].

Sunitinib, a multi-targeted tyrosine kinase inhibitor of VEGF receptors (VEGFRs), PDGFRa/b, KIT, Flt-3 and CSF-1R, is widely used as a first-line therapy for mRCC [8]. Objective response rates of 25–47% have been reported to sunitinib in mRCC [9]. However, disease progression usually occurs about 11 months after the initiation of sunitinib treatment [9]. Furthermore, 10–20% of patients exhibit no clinical benefit [9], [10]. Preclinical studies have identified potential mechanisms behind the development of acquired resistance to anti-angiogenic therapy, including adaptive changes in the activation of VEGF-independent angiogenic pathways, altered cellular metabolism and the activation of cancer stem cells [11–13]. However, the mechanisms of intrinsic resistance, where patients do not benefit at all from VEGF-targeted drugs remain largely unknown [8]. Thus, there is a need for biomarkers predicting sunitinib response and for identifying patients who will benefit from the therapy, but so far no such established markers are in clinical use [14].

Angiopoietin-2 (Ang2, Angpt2) is an endothelial cell-derived growth factor, which binds in an autocrine fashion to the endothelial Tie2 receptor tyrosine kinase on blood and lymphatic vessels [15]. Ang2 is expressed at low levels during normal homeostasis, but at increased levels in many human diseases characterized by increased vascular leak and inflammation, such as sepsis [16], and in cancer, including RCC [17–19]. Pre-clinical studies have demonstrated tumour growth and angiogenesis inhibition using Ang2 blocking reagents, especially, when combined with VEGF-based anti-angiogenic therapies [12,20–23] as well as inhibition of lymph node and distant metastasis [21,24,25]. Angiopoietin antagonists, which are in the clinical development have been combined with paclitaxel in phase III ovarian cancer trials [26], and more recently, with sunitinib in a phase II mRCC trial [27], but more studies are needed to evaluate the benefit of angiopoietin blocking in human cancer.

Ang2 mRNA expression has been reported to predict poor prognosis in breast cancer [28], and high circulating Ang2 levels were reported to predict unfavourable outcome in metastatic colorectal carcinoma [29], mRCC [30] and melanoma [31]. However, few studies have so far directly investigated Ang2 protein expression in human tumour tissues, mainly due to lack of reliable immunohistochemical methods. Thus, the cell type expressing Ang2 in RCC tumour tissues has not been identified and the potential significance of Ang2 protein expression in RCC for tumour angiogenesis, tumour cell proliferation or response to anti-angiogenic therapies remains unknown.

Ki-67 is a large nuclear protein, which has prognostic relevance in many malignant diseases, including local RCC [32,33]. High expression of Ki-67 in patients with local RCC nearly doubled the risk of death [33]. However, Ki-67 has not been largely investigated as a prognostic or predictive factor in mRCC patients treated with angiogenesis inhibitors.

The purpose of this study was to investigate pre-therapeutic Ang2, Ki-67 and CD31 expression in the primary human RCC tissue in patients with metastatic disease, the correlation of baseline Ang2 and Ki-67 expression with tumour vascular content and the relation of their expression to the outcome of first-line sunitinib treatment in patients with mRCC. Using 136 pre-therapeutic samples from primary mRCC tumours, we found that Ang2 is selectively expressed in the tumour vasculature, but not in RCC tumour cells. Baseline Ang2 expression correlated with vascular density, but not with PFS or OS, although high Ang2 expression was associated with clinical benefit to sunitinib. Low expression of Ki-67 correlated significantly with prolonged PFS and OS, suggesting that tumour cell proliferation is a potential prognostic marker for first-line sunitinib therapy in mRCC.

## Materials and Methods

### Patients, Treatment and Assessment of Tumour Response

A total of 181 consecutive patients with mRCC were treated with sunitinib at the Department of Oncology of Helsinki University Central Hospital (HUCH) between October 18, 2006 and May 31, 2012. Of these patients histological specimens could be retrieved from 136 patients, and data about response to sunitinib treatment was available from 126 patients. The main reason for the lack of efficacy data in the remaining ten cases was too short treatment to be evaluable. Patients were identified from the hospital registry and the clinical data were collected from the hospital case records. We required for the study inclusion that the patients had histologically identified mRCC treated with sunitinib as their first systemic treatment for advanced disease. No prior targeted cancer therapies were allowed.

Following the approval of the study protocol by a HUCH Ethics Committee, the formalin-fixed paraffin-embedded samples containing tumour tissue were collected from the archives of the Department of Pathology, HUCH. HUCH ethics committee waived the need for individual consent, since all data were analyzed anonymously, and approved this retrospective study. The cut-off date for the data capture was June 1, 2012. The features of the study patient population are provided in **Table 1**. The median duration of sunitinib treatment was 7.2 months (range 0.23 to 65.3 months).

**Table 1:** Patient Characteristics.

	No. (%)
	(N = 136)
**Sex**	
Male	79 (58)
Female	57 (42)
**WHO performance status**	
0	77 (57)
≥1	59 (43)
**Prior nephrectomy**	135 (99)
**Reason for stopping sunitinib**	
Progression	81 (60)
Adverse events/other	55 (40)
**Heng risk group**	
Favourable	67 (49)
Intermediate	30 (22)
Poor	35 (26)
N.A.	4 (3)
**MSKCC risk group**	
Favourable	22 (16)
Intermediate	81 (60)
Poor	22 (16)
N.A.	11 (8)
**Histological type**	
Clear cell	120 (88)
Papillary	12 (9)
Other	4 (3)

Abbreviations: WHO, World Health Organization; N.A, not available; MSKCC, Memorial Sloan-Kettering Cancer Center

None of the patients had received prior tyrosine kinase inhibitor therapy, but 18 (13%) had received prior interferon-α. Sunitinib was administered until disease progression, death or until toxicity was considered unacceptable. Of the 136 patients, 43 patients had 25 mg/day continuous daily dosing (CDD) as their starting dose, 61 patients had 37.5 mg/day CDD and 32 patients had 50 mg/day 4 weeks on and 2 weeks off, the latter two dosages being widely accepted and used as global standards for the treatment of mRCC [34].

Response to treatment was assessed using computed tomography (CT) performed at 8 to 12 week intervals after starting sunitinib. A professional radiologist interpreted all images. Treatment efficacy was reported according to Response Evaluation Criteria in Solid Tumours v. 1.0 (RECIST).

### Histology and Immunohistochemistry

Five μm thick sections were cut from formalin-fixed, paraffin-embedded tumour tissues and stained using haematoxylin and eosin for histological analysis. Immunohistochemistry (IHC) was performed on deparaffinised (with xylene) and rehydrated sections by using the Tyramide Signal Amplification kit (PerkinElmer, Waltham, MA, USA) following the manufacturer’s instructions. Antigen retrieval was conducted in 10 mM citrate buffer using microwaves at 750 W for 5 min, and subsequently at 450 W for 10 min. Endogenous peroxidase activity and nonspecific binding sites were blocked using 10% hydrogen peroxidase and a TNB (0.5%) blocking buffer (PerkinElmer), respectively. The sections were incubated with goat polyclonal antibodies to amino acid residues Asp68-Phe496 of human Angiopoietin-2 (1:50, 0.2 mg/ml, R&D Systems, Minneapolis, MN) and with a mouse monoclonal antibody to human CD31 (1:100, 0.2 mg/ml, JC70A, DAKO, Glostrup, Denmark), diluted in TNB overnight at +4°C, washed with TNT buffer (100 mM Tris pH 7.4, 150 mM NaCl, 0.05% Tween-20), and subsequently incubated with biotinylated anti-goat or anti-mouse secondary antibodies (1:300, 1.5 mg/ml, Vector Laboratories Inc, Burlingame, CA) in TNB for 30 min at room temperature, washed and detected using a chromogenic visualizing method AEC (3-amino-9-ethylcarbazole) for 8 min. The sections were counterstained with Mayer’s Hemalum Solution (Merck KGaA, Darmstadt, Germany) and mounted using Aquatex (Merck KGaA). To confirm the specificity of the Ang2 staining, Ang2 antibodies were incubated with 5x molar excess of recombinant human Ang2 (3μg) (R&D Systems) in TNB for 20 min, and subsequently used for staining of the tissue sections as above.

Immunostaining for Ki-67 was performed using a Ventana Bench Mark XT immunostainer automate (Ventana Medical Systems, Tucson, Arizona, USA) and a mouse anti-human Ki-67 mAb (MIB-1, DAKO) with a Ventana UltraView Dab V3 (Ventana Medical Systems) kit with amplification.

### Scoring of Ang2, CD31 and Ki-67 Protein Expression

The investigators scored independently the protein expression of Ang2 (JR+AL and PS), CD31 (JR+AL) and Ki-67 (JR+AL and TM). A general consensus was sought whenever the scores differed between the investigators. The mean Ang2+ and CD31+ vessel densities were estimated based on three vascular hot-spot areas using an Olympus Bx50 microscope (Olympus Optical Co., Ltd, Tokyo, Japan) and a 10x objective (magnification x100). The samples were scored for Ang2 and CD31 expression using four expression scores of negative (0), weak (1), moderate (2) and high expression (3). The score 0 was defined as no positive vessels, score 1 as 1–49 vessels, score 2 as 50–99 vessels, and score 3 as ≥100 positively stained vessels per one 10x microscope field. For statistical analysis using the Fisher’s exact test tumours with CD31 expression scores 3, which showed a highly vascular phenotype (≥100 CD31+ vessels/one microscope field), were considered to form the CD31 high category (40% of samples), and the samples with a score 0–2 (<100 vessels/one microscope field) the CD31 low category (60%). Due to the lower level of Ang2 expression, tumours with Ang2 expression scores 2 and 3 (≥50 Ang2+ vessels/one microscope field) and the tumours with Ang2 expression scores 0 and 1 (<50 Ang2+ vessels/one microscope field) were combined to form the Ang2 high (38% of the samples) and Ang2 low categories (62%), respectively. The expression of Ki-67 was considered high when more than 10% of the tumor cell nuclei, measured in three different hot spot areas for each sample, expressed Ki-67 [35]. A pathologist reviewed tumour histology in each case.

Images were captured using a Leica DM LB microscope (Meyer Instruments, Inc, Houston, TX) attached to an Olympus DP50 colour camera using 10x (NA (numerical aperture) 0.25), 20x (NA 0.4) and 40x (NA 0.65) objectives. Magnification was x100, x200 and x400, respectively.

### Statistical Analysis

Progression-free survival (PFS) was calculated according to the Kaplan-Meier method from the date of initiation of sunitinib treatment to the date of documented cancer progression or death, whichever occurred first. Patients alive without progression were censored on the time of the last follow-up visit. Overall survival (OS) was counted from the date of sunitinib initiation to the date or death, censoring patients alive. Survival between groups was compared with the log-rank test. Frequency tables were analysed using the Fisher’s exact test or the χ^2^-test. All *P*-values are two-tailed. The statistical analyses were performed with the IBM SPSS Statistics software for Mac, version 20.0 (IBM Corporation, Armonk, N.Y.).

## Results

### Angiopoietin-2 Expression in the Tumour Vasculature Correlates with Vascular Density

**Fig 1:**
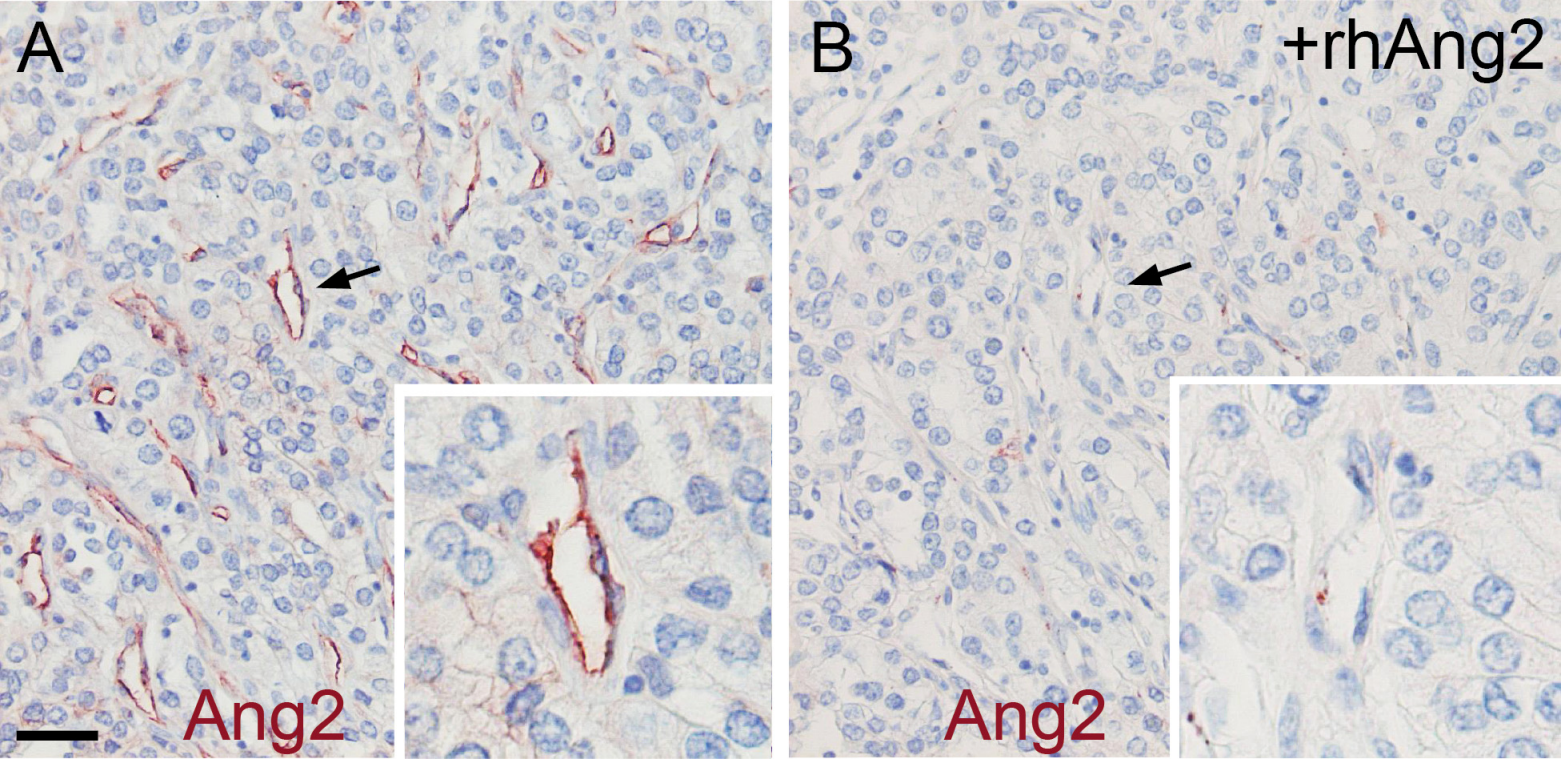
Representative immunohistochemical staining of Ang2 in mRCC. **(A**) immunohistochemical staining of paraffin-embedded mRCC primary tumour tissue using a polyclonal antibody to Ang2. The CD31 expression was scored 3 in the sample. (**B**) An adjacent section was stained using the polyclonal antibody to Ang2, blocked with a 5-fold molar excess of recombinant human Ang2 (+rhAng2). Arrows indicate the magnified areas. Magnifications x200 and x400. Scale bar 40 μm.

The immunohistochemical analysis of primary mRCC tumour tissue showed that Ang2 was specifically expressed in endothelial cells of the tumour blood vessels, but not in the tumour cells. A 5-fold molar excess of recombinant Ang2 was used to neutralize the polyclonal antibody to Ang2. This efficiently abolished the Ang2 signal in the tumour blood vessels confirming the specificity of the staining (**Fig 1**).

**Fig 2:**
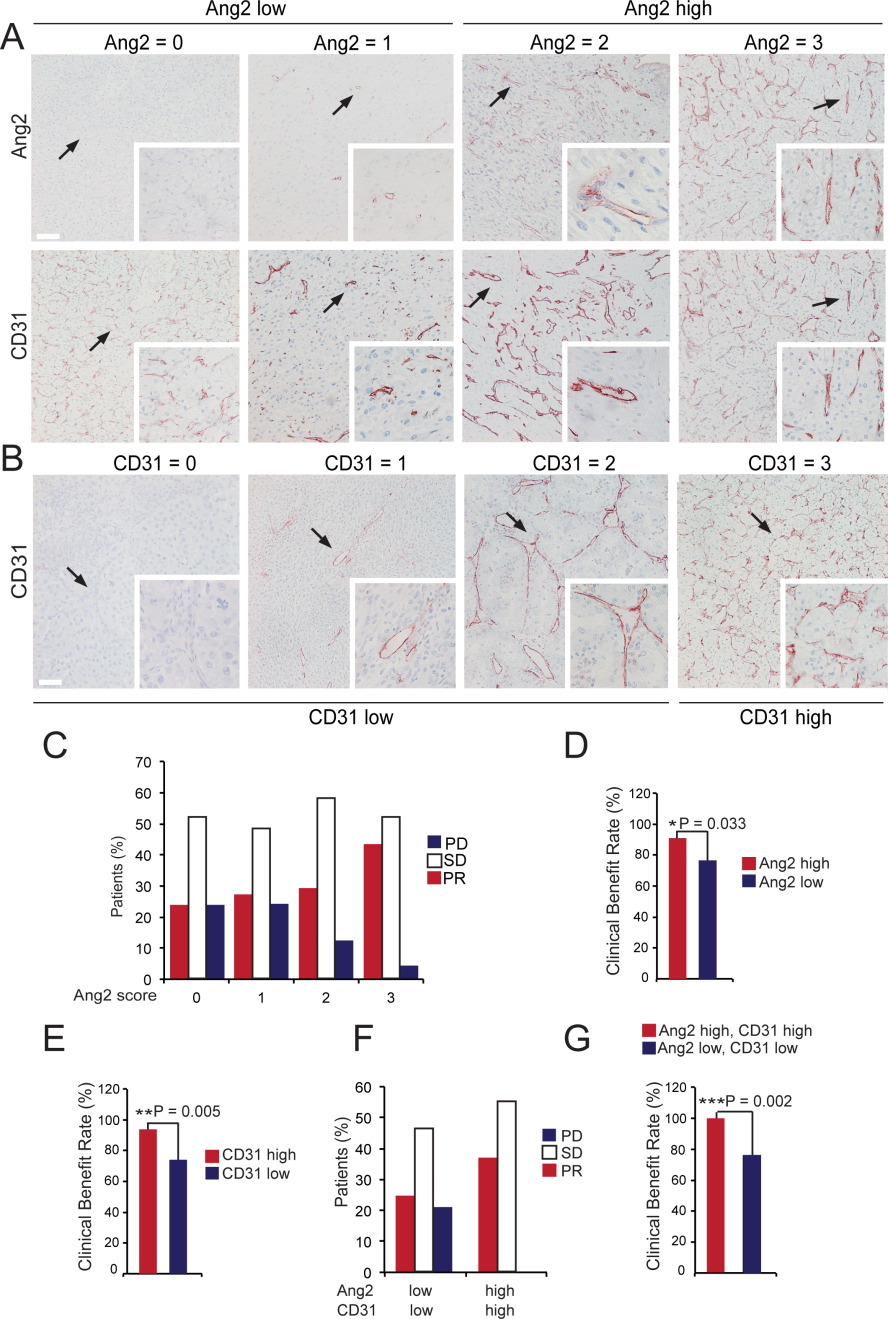
Association of Ang2 and CD31 expression with sunitinib response in mRCC. Immunohistochemical staining of mRCC primary tumour tissue using antibodies to Ang2 (**A**) and CD31 (**A, B**). (**A**) Representative images of mRCC tumours with negative (Ang2 = 0), weak (Ang2 = 1), moderate (Ang2 = 2) and high (Ang2 = 3) Ang2 expression scores, and their distribution in the Ang2 low (scores 0 and 1) and Ang2 high categories (scores 2 and 3) (top panel in A). Adjacent sections were stained for CD31 (bottom panel in A, scores not indicated). (**B**) Representative images of mRCC tumours with negative, weak, moderate (CD31 = 0, CD31 = 1, CD31 = 2) and high (CD31 = 3) CD31 expression scores, and their distribution in the CD31 low (scores 0–2) and CD31 high categories (score 3). (**C**) The percentage of patients with PR, SD and PD responses according to Ang2 expression scores 0–3, from negative (0) to high (3) expression. (**D-E**) High Ang2 (D) and high CD31 (E) protein expression in the mRCC tumour vasculature was associated with increased clinical benefit rate (CBR, PR/SD sunitinib responses) using Fisher’s exact test. (**F**) The percentage of patients with PR, SD and PD responses according to combined Ang2 and CD31 expression. (**G**) The combination of both high Ang2 and high CD31 protein expression was associated with increased CBR responses using Fisher’s exact test. PR, partial response; SD, stabilized disease; PD, progressive disease. Arrows indicate the magnified areas. Magnifications x100 and x400. Scale bar 80 μm.

The analysis of 136 mRCC samples showed consistent Ang2 expression in the tumour vessels, and variation in the levels of Ang2 expression between the samples (ranging from 0 to >100 Ang2 positive vessels/microscopic field, median 5) (**Fig 2A,** S1A and S1C Fig). Occasionally, Ang2 expression was detected in the blood vessels of the glomeruli and in the tubular epithelium in the adjacent normal kidney tissue, and in the blood vessels of the associated fat tissue (not shown).

The tumours, analysed by immunohistochemical staining for CD31, showed variation in the vessel numbers (ranging from 0 to >100 CD31 positive vessels/microscope field, median 80) (Fig 2B, S1B and S1C Fig). The number of patients with expression scores 0, 1, 2 and 3 (representing negative, weak, moderate and high expression) were 50, 35, 27 and 24 for Ang2 and 8, 14, 60, 54 for CD31, respectively. Ang2 expression was correlated with the vascular content of the tumours, when the Ang2 and CD31 expression scores (scores 0–3) where compared (Spearman rank order correlation test, r = 0.47, *P* = 2.3 e-8; S2A Fig). When tumours were further categorized to low and high expression categories according to Ang2 and CD31 expression scores, as explained in the methods, the CD31 high category consisted of 54 (40%) tumours, the CD31 low category of 82 (60%) tumours, Ang2 high category of 51 (38%) tumours, and Ang2 low category of 85 (62%) tumours (S1A and S1B Fig). High cancer Ang2 expression was strongly associated with high CD31 expression when the two distributions were compared (χ^2^-test, *P* <0.0001) (S1 Table).

### High Baseline Endothelial Ang2 and CD31 Expression in the Tumour Are Associated with the Clinical Benefit to Sunitinib

High pre-therapeutic Ang2 expression in the tumour vasculature was associated with a 15-percentage point increase in clinical benefit rate (CBR; partial response (PR) + stable disease (SD)) to sunitinib, when the high and low Ang2 categories were compared (CBR was 91% in the Ang2 high category vs 76% in Ang2 low category, Fisher’s exact test, *P* = 0.033) (Fig 2C and 2D). Only four (9%) patients with high tumour Ang2 expression had progressive disease (PD) as their best response to sunitinib treatment when compared to 19 (24%) patients with low tumour Ang2 expression (*P* = 0.033). In addition, high CD31 expression was associated with a 20-percentage point increase in the CBR to sunitinib, as compared with low CD31 expression (CBR was 94% in the CD31 high category vs. 74% in the CD31 low category, Fisher’s exact test, *P* = 0.005) (Fig 2E). Only three (7%) patients with high CD31 expression had PD as their best response as compared with 20 (25%) patients with low expression of CD31 (*P* = 0.005).

Patients with both high CD31 and high Ang2 expression achieved more frequently clinical benefit with sunitinib as compared to patients with low expression of both of the markers (the CBR was 100% in the Ang2 high and CD31 high category vs. 76% in the Ang2 low and CD31 low category, Fisher’s exact test, *P* = 0.002) (Fig 2F and 2G). None of the patients with high CD31 and high Ang2 expression (n = 29) had progressive disease as their best response as compared with 23 (24%) patients among the rest of the patients (n = 97).

The correlation between Ang2 and CD31 expression and clinical response was analysed based on the four Ang2 expression scores (from negative (0) to high (3) expression) and the three clinical response scores (PR = 1, SD = 2, PD = 3), using the Spearman rank order correlation test. Pre-therapeutic Ang2, but not CD31, expression was correlated with better sunitinib response, especially with Ang2 scores 2 and 3 (Spearman rank order correlation test, *P* = 0.03, S2B Fig), supporting the association of high Ang2 expression with clinical benefit to sunitinib (Fig 2D). In addition, the percentage of Ki-67 positive nuclei correlated with clinical response (Spearman rank order correlation test, *P* = 0.023). The results were visualized in 3-dimensional scatter plots by fitting a linear surface model to the data (S2C and S2D Fig).

When the efficacy analysis was restricted to patients who received either 37.5 or 50 mg sunitinib daily (n = 88), high Ang2 expression and the combination of both high Ang2 and high CD31 expression were similarly associated with a high CBR (Fisher’s exact test, P = 0.040 for the Ang2 high vs. Ang2 low categories, *P* = 0.018 for Ang2 high and CD31 high vs. Ang2 low and CD31 low category). Furthermore, high tumour Ang2 and high CD31 expression were associated with a high CBR also when the analysis was restricted to patients with clear cell RCC (Fisher’s exact test, *P* = 0.003, n = 112).

### Clinical Benefit Rate, but Not Ang2 and CD31 Expression, Is Associated with Prolonged PFS and OS

Median PFS was 8.7 months (95% CI 6.4 to 10.9 months) in the entire series. As expected, achieving a clinical benefit (PR/SD) correlated significantly with longer PFS (median PFS for PR/SD patients 14.1 months and for PD patients 3.3 months, *P* <0.0001) and with longer OS (30.1 months for PR/SD patients vs. 12.7 months for PD patients, *P* <0.0001). However, Ang2 or CD31 expression had no significant association with PFS (median 11.1 months vs. 8.2 months in the Ang2 high vs. Ang2 low categories, respectively, *P* = 0.308; CD31 high vs CD31 low, *P* = 0.851; S2 Table), or with overall survival (22.3 vs. 22.6 months in Ang2 high vs. Ang2 low categories, respectively, *P* = 0.274; 22.3 vs. 22.6 months in the CD31 high and the CD31 low categories, respectively, *P* = 0.949; S2 Table). In line with these results, the duration of SD was not significantly longer in patients with high Ang2 and high CD31 expression (median duration of SD 8.7 months in the Ang2 high and CD31 high category) when compared to patients with low Ang2 and low CD31 expression (median SD duration 10.5 months in the Ang2 low and CD31 low category, *P* = 0.626). Furthermore, there was no statistically significant difference in response to sunitinib, PFS or OS between patient groups receiving 50/37.5 mg vs. 25 mg sunitinib doses (PFS = 9,8 months vs. 7.8 months, P = 0.167; OS = 25.2 months vs. 22.3 months, P = 0.354).

### Low Ki-67 Expression Correlates with Better Outcome

**Fig 3:**
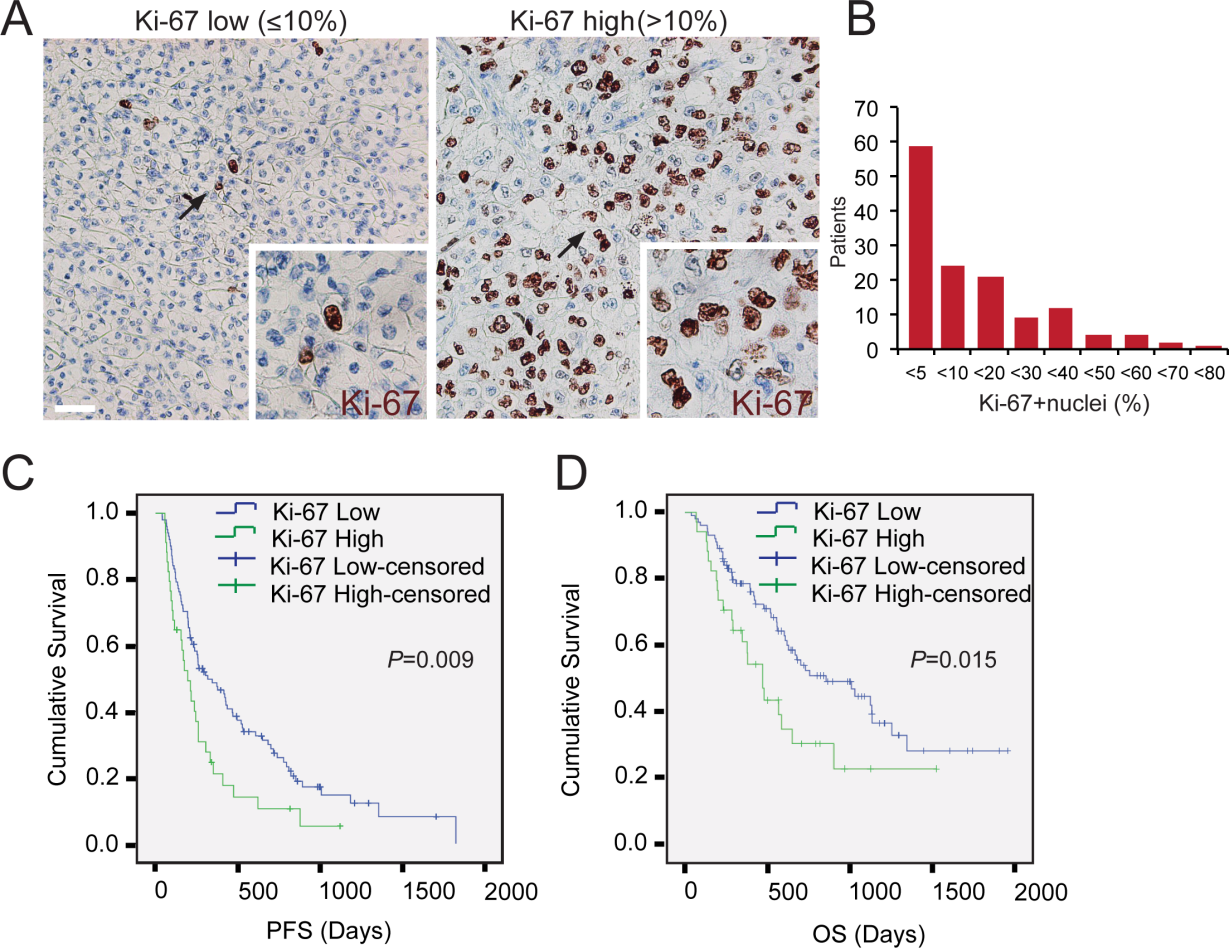
Correlation of Ki-67 expression with progression-free and overall survival in mRCC in response to first-line sunitinib. **(A)** Representative images of tumours with low Ki-67 expression (Ki-67+ nuclei % ≤10%), and high Ki-67 expression (Ki-67+ nuclei % >10%). (**B**) The distribution of patients according to Ki-67 expression, expressed as the % of Ki-67+nuclei. (**C-D**) Kaplan-Meier survival curves (Log rank) of progression-free (C) and cancer specific survival (D) in patients with low Ki-67 (blue) and high (green) Ki-67 expression scores. Arrows indicate the magnified areas. Magnifications x200 and x400. Scale bar 40 μm.

Using 10% of Ki-67 positive nuclei (median 5) as a cut-off, high pre-therapeutic expression of Ki-67 (>10% Ki-67 positive nuclei) was correlated with worse outcome in patients treated with sunitinib (**Fig 3A–3D**). In patients with more than 10% of the tumor cells expressing Ki-67 (25% of the patients), the PFS was 6.5 months, when compared to 10.6 months in patients with low expression of Ki-67 (*P* = 0.009)(Fig 3C). Moreover, high expression of Ki-67 predicted shortened OS in these patients (15.7 months vs. 28.5 months in the high Ki-67 vs. low Ki-67 categories, *P* = 0.015)(Fig 3D).

## Discussion

The mechanisms of both acquired and intrinsic resistance to VEGF-targeted therapy, including sunitinib, remain incompletely understood in human cancer. In addition, biomarkers predicting the clinical response to anti-angiogenic therapy are not yet available. Ang2 is known to promote tumour angiogenesis and metastasis, via signalling pathways not involving the VEGF-VEGF receptor system. Preclinical studies have identified the adaptive increase in Ang2 expression as a potential mechanism behind resistance that develops in response to anti-angiogenic therapy [12,36]. However, little information exists about Ang2 protein expression in human tumours, including RCC, about the cell types that express Ang2 in tumours and the effect of pre-therapeutic tumour Ang2 expression on the clinical response to VEGF-based anti-angiogenic therapy. Our results demonstrate that high baseline Ang2, as well as CD31 expression in the primary RCC tumour vasculature was associated with an initial beneficial response to sunitinib, but not with patient survival. However, high baseline tumour cell proliferation, marked by Ki-67 expression, was strongly associated with poor outcome of patients receiving first-line sunitinib therapy, warranting further analysis of Ki-67 as a potential biomarker of sunitinib therapy in mRCC.

The immunohistochemical method, established in the present study for the detection of Ang2, demonstrated the vascular endothelial cells, but not the RCC tumour cells, as the cell type expressing Ang2, consistently in all of the primary mRCC tumours investigated. Previous studies based on immunohistochemistry have reported Ang2 expression in the tumour cell compartment of many human cancers [37–39] and both in the endothelial and cancer cells in a smaller study based on 45 RCC samples [40]. In colorectal cancer, Ang2 mRNA was detected in the stromal, but not in the tumour cells, however, the stromal cell type expressing Ang2 was not identified [29]. Studies on mouse tumours have suggested endothelial cell-specific Ang2 mRNA expression [41], in line with the Ang2 protein analysis of 136 human mRCC tumours in this study.

Ang2 expression in the vasculature of mRCC tumour tissues, collected prior to initiation of first-line sunitinib therapy, was associated with a beneficial sunitinib response (PR/SD), whereas Ang2 expression was not correlated with OS or PFS in our study. Since patients were treated with first-line targeted therapy, subsequent treatments may have influenced the overall survival of the patients and therefore Ang2 and CD31 expression may not have a direct influence on OS despite the association with initial treatment response. However, achieving a clinical benefit (PR/SD) was significantly associated with longer PFS and OS in the entire patient series, indicating the relevance of using CBR as a measure of sunitinib efficacy. Furthermore, Ang2 and CD31 expression were associated with clinical benefit even when the analysis was restricted to patients receiving the two highest sunitinib doses. Thus, our results support the conclusion that high baseline Ang2 expression in the blood vessels of the primary tumour does not compromise the initial sunitinib response. This result differs from the results of a recent study, which demonstrated that low baseline levels of circulating Ang2 in mRCC patients receiving first-line sunitinib predicted better outcome [30]. The observed differences may arise from the different methods used for measuring Ang2. First, it is not known whether the circulating Ang2 levels correlate with primary tumour-expressed Ang2 in mRCC patients. Alternatively, Ang2 expressed by metastatic nodules, or by the host vasculature as a more general response to disease, might contribute to the determined serum Ang2. Indeed, elevated circulating Ang2 levels have been reported to predict for poor prognosis in numerous non-cancerous diseases, such as sepsis and malaria [16,42–44]. In these diseases, the activated endothelial cells of the normal vessels likely secrete Ang2 [45]. Second, the immunohistochemical method likely detects Ang2 stored in the Weibel-Palade bodies in endothelial cells, which may not correlate with the bioavailable Ang2 pool in the tumours. On the other hand, as Ang2 is expected to function in an autocrine manner on the vascular endothelium, the correlation of the circulating Ang2 with tumour angiogenesis remains to be investigated.

We found that endothelial Ang2 expression in the tumour vasculature correlated with the vascular density of the tumours, as determined by CD31 expression. Few studies have reported increased microvascular density, especially increased number of CD31 positive vessels, as a marker of poor prognosis in untreated ccRCC [46,47], whereas others have found that high CD31 predicts prolonged overall survival in RCC [48]. Dornbusch et al. showed that in metastatic RCC treated with sunitinib, high CD31 expression compared to low CD31 in the primary tumour was associated with better sunitinib response [49], in line with our results of the association of high CD31 with high clinical benefit rate. In addition, Puerto-Nevado et al. demonstrated using immunohistochemistry that high phospho-VEGFR2 in the tumour stroma, correlated with prolonged survival (PFS and OS) of mRCC patients treated with sunitinib [50]. These results suggest that angiogenic tumor vasculature characterized by high phospho-VEGFR-2 and Ang2 signaling is associated with beneficial sunitinib response [51].

The immunohistochemical analysis of large tumour sections in this study revealed, in the majority of the cases, intratumoural heterogeneity of the number of tumour blood vessels, Ang2 expression in the vessels and tumour cell proliferation, which is in line with intratumoural heterogeneity previously observed in mRCC [52]. Therefore, estimates for the overall vascular density, the Ang2 positive vessels counts and Ki-67 expression, were based on several areas per each tumour sample. However, we cannot exclude the effects of intratumoural heterogeneity on our results, although the large tumour sections used in this study likely provide a better estimate of overall tumour parameters than tumour microarrays.

Our results demonstrated that high baseline expression of Ki-67 predicted poor survival in mRCC patients receiving first-line sunitinib. Previously, high Ki-67 has been found to predict poor survival in local RCC, and correlate with the outcome in mRCC patients receiving immunotherapy [33,35]. Our results are in line with an analysis of sequential tumour samples obtained during sunitinib and pazopanib treatment of mRCC patients, which demonstrated that therapy-associated increase in Ki-67 expression correlated with poor prognosis [53].

We have analysed a relatively homogenous patient population of a moderate size consisting of consecutive patients from one academic institute. 68% of the patients received globally accepted dosing schedules and daily doses of sunitinib (37.5–50 mg/day), representing a typical non-trial patient population [34]. The main findings in our study were that Ang2 was specifically expressed in the tumour vasculature, but not in RCC tumour cells, Ang2 expression in the primary tumours correlated with tumour vascular density and that the high baseline endothelial Ang2 levels were associated with an initial beneficial response to first-line sunitinib. However, Ang2 was not associated with PFS and OS, whereas low Ki-67 was significantly associated with longer PFS and OS in patients receiving first-line sunitinib. An analysis of a different patient cohort is warranted to confirm the results obtained in this retrospective series. In conclusion, the results suggest that baseline tumour cell proliferation in the primary tumour may be considered as a potential biomarker for sunitinib efficacy, and warrants further study in series of patients treated with other anti-angiogenic agents.

## Supporting Information

S1 Fig Distribution of patients according to Ang2 and CD31 expression scores. (**A-B**) The distribution of patients (n = 136) according to the Ang2 (A) and CD31 (B) expression scores (from negative (0) to high (3) expression), and further categorisation into the low and high expression categories. (**C**) The distribution of patients according to both Ang2 and CD31 expression scores.
(PDF)

S2 Fig Correlation of Ang2, CD31, Ki-67 and response scores. (**A**) Spearman rank order correlation between Ang2 and CD31 expression scores, and (**B**) Ang2 expression and response scores. Boxplots alongside the xy-axis represent the distribution of samples based on Ang2, CD31 and response scores. Ang2 scores are distributed primarily towards the lower scores (0, 1, 2), whereas the CD31 score distribution is towards scores 2 and 3. (A) There is a positive correlation between Ang2 and CD31 expression (Spearman rank order correlation test, r = 0.47, *P* = 2.3 e-8). (B) Ang2 expression correlates with better sunitinib response (Spearman rank order correlation test, *P* = 0.03). Green line, overall correlation; red line, locally adjusted Loess correlation; green dot, mean; green ellipses, 5%, 25% and 50% data concentrations; dotted red lines, 95% confidence intervals. For clarity of the data concentrations, a small random jitter is added to visualize the data points. (**C-D**) 3-dimensional scatter plots with linear fitted surface (purple) of the Ang2, Ki-67 and response scores (C), and of Ang2, CD31 and response scores (D) with a smoothed linear least-squares surface fitting (green) for the data, created using the R package. The plots indicate the influence of decreased Ki-67 (C) and increased Ang2 expression (C-D) towards better response (lower response scores) as a decline in the purple surface towards low Ki-67+ nuclei % (C) and Ang2 expression score 3 (C-D). Response scores: PR = 1, SD = 2, PD = 3; Ang2 scores 0–3; CD31 scores 0–3; from negative (0) to high (3) expression.
(TIF)

S1 File Supplementary Information. (PDF)

S1 Table Distribution of patients according to tumour Ang2 and CD31 expression scores in the Ang2 and CD31 high and low categories. (PDF)

S2 Table Associations between renal cell cancer Ang2 and CD31 expression and objective response rate, progression-free survival (PFS) and overall survival (OS). (PDF)
