# Supplementary material for: Association of Angiopoietin-2 and Ki-67 Expression with Vascular Density and Sunitinib Response in Metastatic Renal Cell Carcinoma
Source: PLoS One. 2016 Apr 21;11(4):e0153745. doi: 10.1371/journal.pone.0153745 (PMC4839598; doi:10.1371/journal.pone.0153745)
Supplement: S1 Fig — (A-B) The distribution of patients (n = 136) according to the Ang2 (A) and CD31 (B) expression scores (from negative (0) to high (3) expression), and further categorisation into the low and high expression categories. (C) The distribution of patients according to both Ang2 and CD31 expression scores. (PDF) [file pone.0153745.s001.pdf]

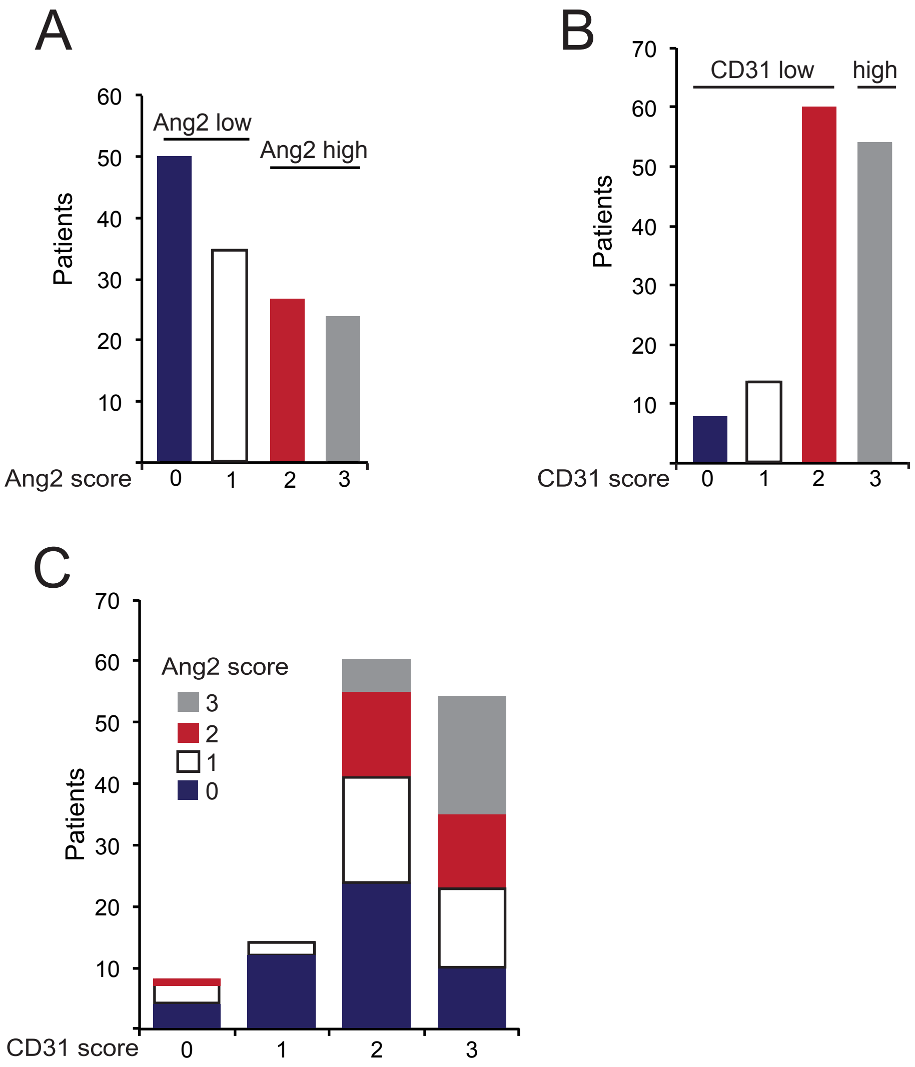

S1 Fig. Distribution of patients according to Ang2 and CD31 expression scores.

Rautiola, Lampinen et al.
