# Supplementary figures and images for: Association of Angiopoietin-2 and Ki-67 Expression with Vascular Density and Sunitinib Response in Metastatic Renal Cell Carcinoma
Source: PLoS One. 2016 Apr 21;11(4):e0153745. doi: 10.1371/journal.pone.0153745 (PMC4839598; doi:10.1371/journal.pone.0153745)

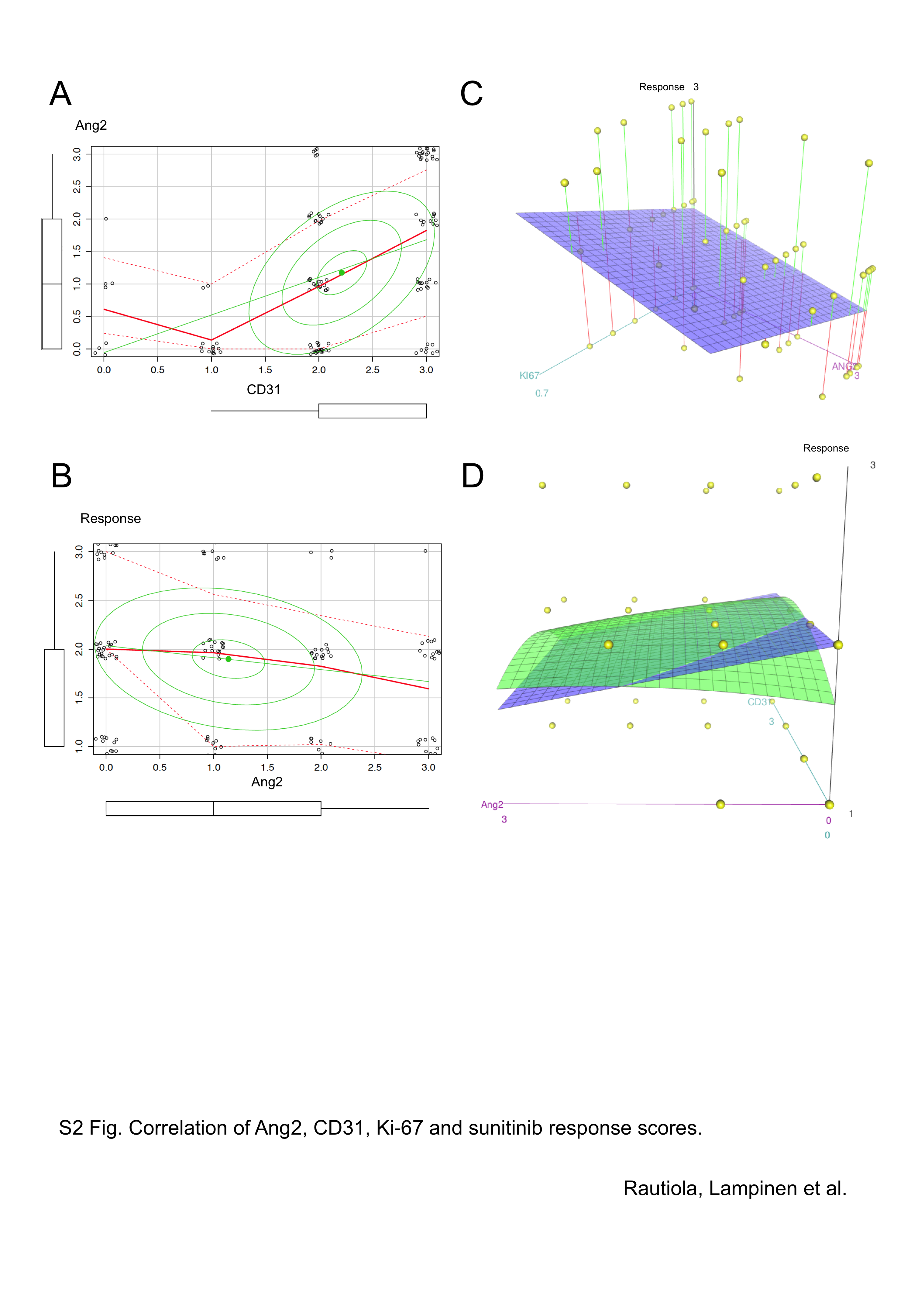

Supplement: S2 Fig — (A) Spearman rank order correlation between Ang2 and CD31 expression scores, and (B) Ang2 expression and response scores. Boxplots alongside the xy-axis represent the distribution of samples based on Ang2, CD31 and response scores. Ang2 scores are distributed primarily towards the lower scores (0, 1, 2), whereas the CD31 score distribution is towards scores 2 and 3. (A) There is a positive correlation between Ang2 and CD31 expression (Spearman rank order correlation test, r = 0.47, P = 2.3 e-8). (B) Ang2 expression correlates with better sunitinib response (Spearman rank order correlation test, P = 0.03). Green line, overall correlation; red line, locally adjusted Loess correlation; green dot, mean; green ellipses, 5%, 25% and 50% data concentrations; dotted red lines, 95% confidence intervals. For clarity of the data concentrations, a small random jitter is added to visualize the data points. (C-D) 3-dimensional scatter plots with linear fitted surface (purple) of the Ang2, Ki-67 and response scores (C), and of Ang2, CD31 and response scores (D) with a smoothed linear least-squares surface fitting (green) for the data, created using the R package. The plots indicate the influence of decreased Ki-67 (C) and increased Ang2 expression (C-D) towards better response (lower response scores) as a decline in the purple surface towards low Ki-67+ nuclei % (C) and Ang2 expression score 3 (C-D). Response scores: PR = 1, SD = 2, PD = 3; Ang2 scores 0–3; CD31 scores 0–3; from negative (0) to high (3) expression. (TIF) [file pone.0153745.s002.tif]
