# Supplementary material for: Association of Angiopoietin-2 and Ki-67 Expression with Vascular Density and Sunitinib Response in Metastatic Renal Cell Carcinoma
Source: PLoS One. 2016 Apr 21;11(4):e0153745. doi: 10.1371/journal.pone.0153745 (PMC4839598; doi:10.1371/journal.pone.0153745)
Supplement: S1 File — (PDF) [file pone.0153745.s003.pdf]

Supporting Information for the manuscript

Association of Angiopoietin-2 and Ki-67 expression with vascular density and sunitinib response in metastatic renal cell carcinoma

Juhana Rautiola[1¶], Anita Lampinen[2¶], Tuomas Mirtti[4,5], Ari Ristimäki[5], Heikki Joensuu[1], Petri Bono[1] and Pipsa Saharinen[2,3*]

[1]Comprehensive Cancer Center, Helsinki University Hospital, P.O.B. 180, 00029 HUS, Finland and University of Helsinki, Finland

[2]Translational Cancer Biology Program, Research Programs Unit, and Department of Virology, Haartman Institute, Biomedicum Helsinki, Haartmaninkatu 8, P.O.B. 63, FI-00014 University of Helsinki, Finland

[3]Wihuri Research Institute, Biomedicum Helsinki, Haartmaninkatu 8, FI-00290 Helsinki, Finland

[4]Institute for Molecular Medicine Finland, Haartmaninkatu 8, P.O.B. 63, FI-00014 University of Helsinki, Finland

[5]Pathology, Research Programs Unit and HUSLAB, University of Helsinki and Helsinki University Hospital, P.O.B. 400, FI-00029 HUS, Helsinki, Finland

***Corresponding author:** Pipsa Saharinen, E-mail: Pipsa.Saharinen@Helsinki.fi

[¶]These authors contributed equally.

Supporting Information

Non-parametric Spearman rank order correlation test was used to analyse the correlation between Ang2 and CD31 expression scores (Ang2 scores 0-3, CD31 scores 0-3, from negative (0) to high (3) expression) and the response scores (response scores 1-3, PR=1, SD=2, PD=3) using the R-package. All statistical tests were two-sided. Linear fitted surface modeling [1] and smoothed linear least-squares surface fitting [2] were adopted to visualize the correlation of the Ang2, CD31 and Ki-67 expression and sunitib response scores using the scatter 3D function in the car package of the R program.

References

1. Fox J and Weisberg S. An {R} Companion to Applied Regression, Second Edition. Thousand Oaks CA: Sage. (2011) URL: http://socserv.socsci.mcmaster.ca/jfox/Books/Companion.

2. R Core Team (2015). R: A language and environment for statistical computing. R Foundation for Statistical Computing, Vienna, Austria. URL http://www.R-project.org/).
