# Supplementary material for: Association of Angiopoietin-2 and Ki-67 Expression with Vascular Density and Sunitinib Response in Metastatic Renal Cell Carcinoma
Source: PLoS One. 2016 Apr 21;11(4):e0153745. doi: 10.1371/journal.pone.0153745 (PMC4839598; doi:10.1371/journal.pone.0153745)
Supplement: S1 Table — (PDF) [file pone.0153745.s004.pdf]

	CD31 high	CD31 low
Ang2 high	31	20
Ang2 low	23	62

S1 Table. Distribution of patients according to tumour Ang2 and CD31 expression scores in the Ang2 and CD31 high and low categories.
