# Supplementary material for: Association of Angiopoietin-2 and Ki-67 Expression with Vascular Density and Sunitinib Response in Metastatic Renal Cell Carcinoma
Source: PLoS One. 2016 Apr 21;11(4):e0153745. doi: 10.1371/journal.pone.0153745 (PMC4839598; doi:10.1371/journal.pone.0153745)
Supplement: S2 Table — (PDF) [file pone.0153745.s005.pdf]

	All patients	Ang2 high	Ang2 low	*P*	CD31 high	CD31 low	*P*
PR (%)	37	17	20	0.196	16	21	0.518
SD+PD (%)	89	30	59		33	56	
PFS (months)	8.7	11.1	8.2	0.308	8.7	8.6	0.851
OS (months)	22.6	22.3	22.6	0.274	22.3	22.6	0.949

S2 Table. Associations between renal cell cancer Ang2 and CD31 expression and objective response rate, progression-free survival (PFS) and overall survival (OS).
